# Comprehensive analysis of histone post-translational modifications in mouse and human male germ cells

**DOI:** 10.1186/s13072-016-0072-6

**Published:** 2016-06-21

**Authors:** Lacey J. Luense, Xiaoshi Wang, Samantha B. Schon, Angela H. Weller, Enrique Lin Shiao, Jessica M. Bryant, Marisa S. Bartolomei, Christos Coutifaris, Benjamin A. Garcia, Shelley L. Berger

**Affiliations:** Department of Cell and Developmental Biology, University of Pennsylvania, Philadelphia, PA 19104 USA; Department of Biochemistry and Biophysics, University of Pennsylvania, Philadelphia, PA 19104 USA; Department of Reproductive Endocrinology and Infertility, Obstetrics and Gynecology, University of Pennsylvania, Philadelphia, PA 19104 USA; Epigenetics Program, University of Pennsylvania, Philadelphia, PA 19104 USA; Biomedical Sciences Graduate Program, Perelman School of Medicine, University of Pennsylvania, Philadelphia, PA 19104 USA; Institute Pasteur, 75724 Paris, France

**Keywords:** Epigenetics, Sperm, Testes, Histone, Post-translational modifications, Male germ cells, Spermiogenesis, Paternal epigenetics, Fertility

## Abstract

**Background:**

During the process of spermatogenesis, male germ cells undergo dramatic chromatin reorganization, whereby most histones are replaced by protamines, as part of the pathway to compact the genome into the small nuclear volume of the sperm head. Remarkably, approximately 90 % (human) to 95 % (mouse) of histones are evicted during the process. An intriguing hypothesis is that post-translational modifications (PTMs) decorating histones play a critical role in epigenetic regulation of spermatogenesis and embryonic development following fertilization. Although a number of specific histone PTMs have been individually studied during spermatogenesis and in mature mouse and human sperm, to date, there is a paucity of comprehensive identification of histone PTMs and their dynamics during this process.

**Results:**

Here we report systematic investigation of sperm histone PTMs and their dynamics during spermatogenesis. We utilized “bottom-up” nanoliquid chromatography–tandem mass spectrometry (nano-LC–MS/MS) to identify histone PTMs and to determine their relative abundance in distinct stages of mouse spermatogenesis (meiotic, round spermatids, elongating/condensing spermatids, and mature sperm) and in human sperm. We detected peptides and histone PTMs from all four canonical histones (H2A, H2B, H3, and H4), the linker histone H1, and multiple histone isoforms of H1, H2A, H2B, and H3 in cells from all stages of mouse spermatogenesis and in mouse sperm. We found strong conservation of histone PTMs for histone H3 and H4 between mouse and human sperm; however, little conservation was observed between H1, H2A, and H2B. Importantly, across eight individual normozoospermic human semen samples, little variation was observed in the relative abundance of nearly all histone PTMs.

**Conclusion:**

In summary, we report the first comprehensive and unbiased analysis of histone PTMs at multiple time points during mouse spermatogenesis and in mature mouse and human sperm. Furthermore, our results suggest a largely uniform histone PTM signature in sperm from individual humans.

**Electronic supplementary material:**

The online version of this article (doi:10.1186/s13072-016-0072-6) contains supplementary material, which is available to authorized users.

## Background

Mammalian spermatozoa are highly specialized, exhibiting a unique nuclear and chromatin structure that packages the paternal genome to minimize DNA damage and promote efficient navigation of the female reproductive tract to fertilize the oocyte. During spermatogenesis, the nuclear organization and chromatin structure of male germ cells undergo a dramatic and highly unique reorganization. This initiates with meiotic division of diploid male spermatogonia to produce four haploid round spermatids, which then undergo spermiogenesis, comprising compaction and condensation, cytoplasmic shedding, acrosome formation, and nuclear elongation. During these stages, nucleosome acetylation is increased in round and elongating spermatids, which relaxes and increases accessibility of chromatin to facilitate histone eviction [1, 2]. A hallmark of mammalian sperm is the highly compact and condensed structure of chromatin, in which depending on the species, approximately 90–99 % of histones are replaced by small, basic, arginine-rich proteins termed protamines [3].

The histone complement retained in mature sperm is critically important, as protamine-deficient mice are infertile [4] and abnormal protamine incorporation and histone retention have been linked to human infertility [5–9]. Furthermore, paternally inherited sperm histones are incorporated into the zygotic chromatin [10]. However, the specific genomic locations of the small fraction of retained histones in mature sperm have been controversial [11]. Clearly, a full understanding of the paternal epigenome is important from a clinical and translational perspective, as increasing numbers of couples utilize assisted reproductive technologies (ART), including *in vitro* fertilization (IVF) and intracytoplasmic sperm injection (ICSI), to conceive children.

Histone proteins are a key component of chromatin and act as the structural unit for packaging of DNA. An octamer of two copies each of the four core histones (H2A, H2B, H3, and H4) forms the fundamental unit of chromatin termed the nucleosome, around which wraps 147 bp of DNA [12]. In addition to the canonical core histones, the linker histone H1 aids in shielding the negatively charged DNA between nucleosomes, and multiple non-canonical histone isoforms have been identified. Each histone is composed of a large globular domain that interacts with the DNA and an amino-terminal tail that protrudes from the core of the nucleosome. Covalent addition of post-translational modifications (PTMs, e.g., phosphorylation, ph; acetylation, ac; methylation, me; crotonylation, cr) to histone amino acid residues occurs, prominently on the amino terminus, although the carboxyl terminus and globular domains are also decorated with PTMs [13]. PTMs are associated with cellular and physiological processes, including transcriptional activation and silencing, repression of repetitive elements, DNA repair, enhancer licensing, cell differentiation, and regulation of disease [14, 15]. Specific histone PTMs are present during the individual stages of spermatogenesis, including primordial germ cell differentiation [16], meiotic recombination [17, 18], spermiogenesis [19, 20], and ultimately, marking the retained histones in mature sperm [21–25]. However, to date, these studies have analyzed individual PTMs, typically utilizing antibodies during a single stage of male germ cell development. With the exception of recent analyses of mature mouse sperm [25], a comprehensive and unbiased analysis of the histone PTM complement in multiple stages of mouse spermatogenesis has not been reported.

**Fig. 1:**
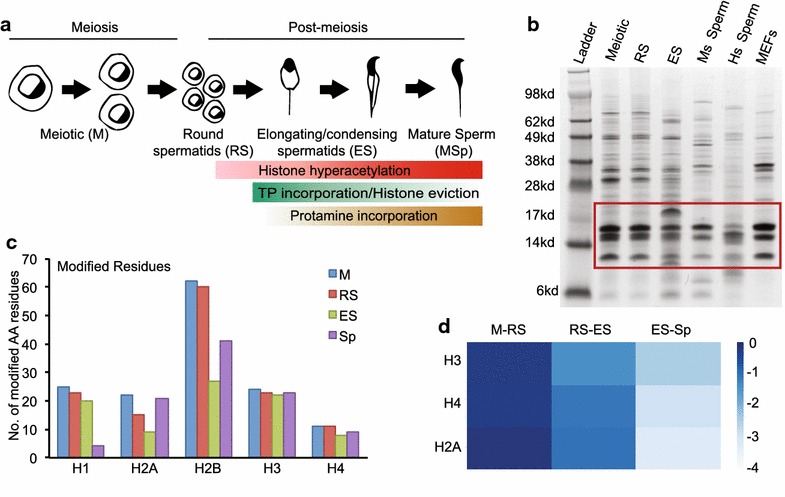
Schematic overview and validation of histone post-translational modifications identified during mouse spermatogenesis. **a** Diagram of spermatogenesis and associated changes in chromatin composition. **b** Coomassie-stained protein gel of acid-extracted proteins in meiotic, round spermatids (RS), elongating spermatids (ES), mature mouse sperm (Ms Sperm), human (Hs) sperm, and mouse embryonic fibroblasts (MEFs). Histones portion is located inside *red box*. **c** Total number of modified amino acid residues on all histone isoforms. **d** Heatmap depicting the fold changes in total canonical histones for each stage of spermatogenesis, i.e., M to RS, RS to ES, and ES to Sp

Recent advances in mass spectrometry (MS) allow for high-resolution identification and analysis of individual and combinatorial PTM patterns [26]. Specifically, “bottom-up” nanoliquid chromatography–tandem mass spectrometry (nano-LC-MS/MS) involves cleavage of histones into specific peptides with known sequences that are analyzed based on charge and mass for the presence of covalently attached PTMs. Hence, this provides an unbiased identification of histone PTMs without specific antibodies. Given the unique progression of spermatogenesis that encompasses a range of processes driven by profound chromatin changes (including meiosis, repair of programmed DNA double-strand breaks during recombination, and histone eviction), our aim is to create a comprehensive characterization of histone PTMs during mouse spermatogenesis (Fig. 1a), including in meiotic spermatocytes, round spermatids, elongating/condensing spermatids, and mature sperm.

Furthermore, given the clinical and translational importance of the paternal epigenome and potential links to infertility, we utilized nano-LC-MS/MS on histones from human sperm to create a comprehensive profile of histone PTMs in clinically defined normal sperm (WHO criteria 5th edition [27]) to determine whether, first, there is uniformity of relative abundance of histone PTM across multiple human individuals and, second, whether PTMs are conserved between mouse and human. Overall, defining histones PTMs in human and mouse sperm, and during the multiple stages of spermatogenesis, is of enormous interest and importance to reproductive biology and fundamental epigenetic study.

## Results

### Identification of histone isoforms and modifications during mouse spermatogenesis

We performed a broad and unbiased analysis to determine identities and relative abundance of histone PTMs during mouse spermatogenesis using “bottom-up” nanoliquid chromatography–tandem mass spectrometry (nano-LC-MS/MS). We isolated meiotic (M), round spermatids (RS), elongating/condensing spermatids (ES), and mature sperm (Sp, Fig. 1a) of adult wild-type SV129 male mice. Following STAPUT velocity sedimentation of testis cell separation [28] and collection of mature sperm from the epididymis, the histones were enriched by acid extraction (red box, Fig. 1b). “Bottom-up” nano-LC-MS/MS proteomic analysis was performed on trypsin-digested peptides, allowing for sensitive detection of individual histone PTMs, and combinatorial PTMs within a single cleaved peptide. Initial identification of peptides present in M, RS, ES, and Sp was conducted with the Mascot search engine by comparing peptide sequences to a database including all mouse histone sequences. In all cell types, all four core histones (H3, H4, H2A, and H2B) and the linker histone H1 were identified (Table 1). In addition, peptides were identified from ten H2A, ten H2B, two H3, and seven H1 isoforms, including the testis-specific TH2B and H1t isoforms (Table 1).

**Table 1:** Histone isoforms and post-translational modifications (PTMs) identified during mouse spermatogenesis and in mature mouse sperm

*Histone isoforms*	
H1	H1.0, H1.1, H1.2, H1.4, H1.5, H1.t, HILS
H2A	H2A.1, H2A.1h, H2A.2a, H2A.2b, H2A.2c, H2A.3, H2A.J, H2A.V, H2A.X, H2A.Z
H2B	tH2B, H2B.1b, H2B.1h, H2B.1k, H2B.1m, H2B.1p, H2B.2b, H2B.2e, H2B.3a, H2B.3b
H3	H3, H3.1, H3.3
H4	H4
*Histone post*-*translational modifications*	
M	
H1	K16ac, K21me1, S57ph, K63me
H1t	M58ox, S59ph, K64me2, K65me3, K76ac, R80me1, S140ph, K142ac, K145me1/3, T146ph, K147cr, K150ac, K151me1, K170ac, S177ph, K190me1, K192cr, M193ox, M195ox
H2A	K5ac, K9ac, R35me1, K36ac, K74ac, R77me1, K125ac, K129me1
H2AV/Z	K11cr, K11me2, K13cr, K13me2/3, K15ac, K15me3, S18ph, R19me1
H2B	S4ph^b^, K5ac, K5me1/2/3, S6ph, K11ac, K11cr, K11me1/2/3, K12ac, K12cr, K12me1/3, S14ph, K15ac, K15cr, K15me1/3, K16ac, K16cr, K16me1/3, K20ac, K24me3, K27me1, K27cr, K28me3, R29me1, K120me2, S123ph, K125me3
tH2B	K6ac, K6cr, K6me1/3, K12ac, K12me1/3, K13cr, K13me1, K16ac, K16cr, K16me1/3, K17cr, K21ac, K24ac, K25me1
H3	K4me1, K9me1/2/3, K14ac, K23me1/2, R26me, K27me1/2/3, K36ac, K36me1/2/3, K37me3, R53me1, K56ac, K79me1/2, M120ox
H4	K5ac, K8ac, K12ac, K12me1, K12cr, K16ac, K16cr, R17me1, K20me1/2/3, K31ac, R35me1, M84ox, K91ac, R92me1
RS	
H1	K16ac, *S40ph*, S57ph, K63me1
H1t	M58ox, S59ph, K64me1/3, K76ac, R80me1, S140ph, K142ac, *S144ph*, K145me2/3, T146ph, *K147ac*, K147cr, *K147me1*, K150ac, K170ac, S177ph, M193ox, M195ox
H2A	K5ac, R35me1, K36ac, K125ac, K129me1
H2Z	*K4ac*, K11cr, K11me2, K13cr, K13me2, *T14ph*, K15me3, K15ac, R19me1
H2B	S4ph ^b^, K5ac, *K5cr*, K5me1/2/3, *R5me* ^a^, K11ac, K11me1/3, K12ac, K12cr, K12me1/2/3, S14ph, K15ac, K15cr, K15me1/2/3, K16ac, K16cr, K16me1/3, K20ac, K24me3, K27cr, *K28ac*, *K28cr*, R29me1, K120me2, S123ph, K125me3
tH2B	K6ac, K6me1, S11ph, K12me,1 *K13ac*, K13me1/3, K16ac, K16me1, K16cr, *K17me1*, *K21ac*
H3	K4me1, K9me1/2/3, K14ac, *K18me1/2*, K23me1/2, R26me1, K27me1/2/3, K36me1/2/3, R53me1, K56ac, K79me1/2, M120ox
H4	K5ac, K8ac, K12ac, K12me1, K16ac, *K16me1*, R17me1, K20me1/2/3, K31ac, R35me1, M84ox, K91ac, R92me1
ES	
H1	K16ac, S57ph, K63me1, *K74ac*, *R78me1*
H1t	M58ox, K76ac, R80me1, S140ph, K142ac, T146ph, K147me1, K147cr, *K151me3*, K170ac, M193ox, M195ox
H2A	K5ac, *K74ac*, *K75ac*, *R77me1*
H2AZ	K11me2, K13me2, T14ph, K15ac, K15me3, R19me1
H2B	K5ac, K5cr, K5me1, K11ac, K11me1/2, K12me1/3, K15cr, K15me1, K16ac, K16cr, K16me1, K120me2, S123ph, K125me3
tH2B	K6ac, K12me3, K13me1, K16me1, K17me1, K21ac
H3	K9me1/2/3, K14ac, *K23ac*, K23me1, R26me1, K27me1/2/3, *K36ac*, K36me1/2, *K37me3*, R53me1, K56ac, K79me1/2, M120ox
H4	K5ac, K8ac, K12ac, K16ac, K20me1/2/3, M84ox, K91ac, R92me1
Sp	
H1	*K19ac* ^c^, *K25me1*, *K27me1* ^c^, *M30ox* ^c^
H2A	*M51ox*, K74ac, K75ac, R77me1
H2AV/Z	*K7ac*, *K11cr*, K11me2, *K13cr*, K13me1/2, T14ph, *K15cr*, K15me1/3, *S18ph*, *K74me3*, *K77cr*, *K79cr*
H2B	K5ac, K5cr, K5me1/2, *S6ph*, K11ac, K11me1/3, *K12ac*, K12cr, K12me1/3, *S14ph*, *K15ac*, K15cr, K15me1/3, K16ac, K16cr, K16me1, *K24me3*, *K27cr*, *K27me1*, *K28ac*, *K28cr*, *K28me3*, *R29me1*, K120me2, S123ph, K125me3
H3	K9me1/2/3, K14ac, K23ac, R26me1, K27me1/2/3, K36ac, K36me1/2, K37me3, *K37cr*, *R40me1*, R53me1, K56ac, K79me1/2, M120ox
H4	K5ac, K8ac, K12ac, K16ac, *R17me1*, K20me1/2/3, M84ox, K91ac, *K91cr*, R92me1

Includes PTMs identified on any isoform except those with modified, non-conserved amino acid residues (H1t, H2AV, H2AZ, tH2B). Underlined PTMs do not appear in the subsequent cell stage, and italicized PTMs were not present in the previous cell stage

^a^R5 is present only on the H2B3A isoform

^b^S4 is present only on H2B3B isoform

^c^Present only on H1.0 isoform

Bioinformatic analysis identified 18 amino acid residues on histone H2A and associated isoforms that were post-translationally modified, while 18, 14, and 11 sites were identified on H2B, H3, and H4 isoforms, respectively (Table 1; see Figs. 4, 5). Eleven amino acid residues were modified on the amino terminus and globular domains of non-testis-specific histone H1 isoforms, while 20 residues were modified on the testis-specific H1t isoform (Table 1). Interestingly, this highly modified region of H1t was only detected from M, RS, and ES stages, whereas no modifications were found on H1t from mature mouse sperm (Table 1). In general, total modified amino acid residues on histone H1 decreased slightly from the meiotic to ES stage prior to a dramatic loss of nearly all modified amino acids in mature sperm (Fig. 1c). Interestingly, the number of modified amino acid residues widely varied by histone and cell type. The total number of modified residues decreased on histones H2A and H2B through the ES stage, prior to an increase of PTMs in sperm, while the number of modified residues remained similar on histones H3 and H4 at all stages of spermatogenesis (Fig. 1c; Table 1). The most commonly identified PTMs were acetyl and monomethyl groups, appearing frequently on lysine (K) residues. Di- and trimethylated K residues, and crotonyl, phosphoryl, and oxidative groups were also identified (Table 1). A complete list of PTMs identified in mouse M, RS, ES, and Sp cells is located in Table 1. Due to the inability to differentiate the exact histone isoform on which some PTMs were located, we have presented and analyzed the data in this manuscript as the compilation of PTMs present on any highly conserved isoform (i.e., H1.0, H1.1, H1.2, H1.4). However, as some histone isoforms have amino acid insertions and sequence variance (H1t, H2AV, and Z), we have listed the identified PTMs for these isoforms individually.

### Relative abundance of histone PTMs during spermatogenesis

To obtain insight into quantitative changes in histone PTMs identified in the initial database search, we used a label-free approach to estimate the area under the curve for the relative abundance of specific unmodified and modified peptides based on the full MS spectra. The abundance of each modified peptide was then normalized by the total amount of peptide, to determine the relative abundance of each modification in relation to the sum of all peptides (total amount of modified and unmodified peptide = 100 %). We then examined changes in the relative abundance of histone PTM between stages of spermatogenesis by calculating the fold change in relative abundance from each subsequent stage (that is, M to RS, RS to ES, and ES to Sp). As expected, total core histone abundance, determined by the quantification of previously validated histone peptides [29], decreased throughout spermatogenesis, with the highest amount detected in meiotic cells and lowest in Sp (Fig. 1d). Hence, based on the overall reduced histone abundance, we normalized individual histone PTMs to total histone content to adjust for loss in maturing male germ cells.

**Fig. 2:**
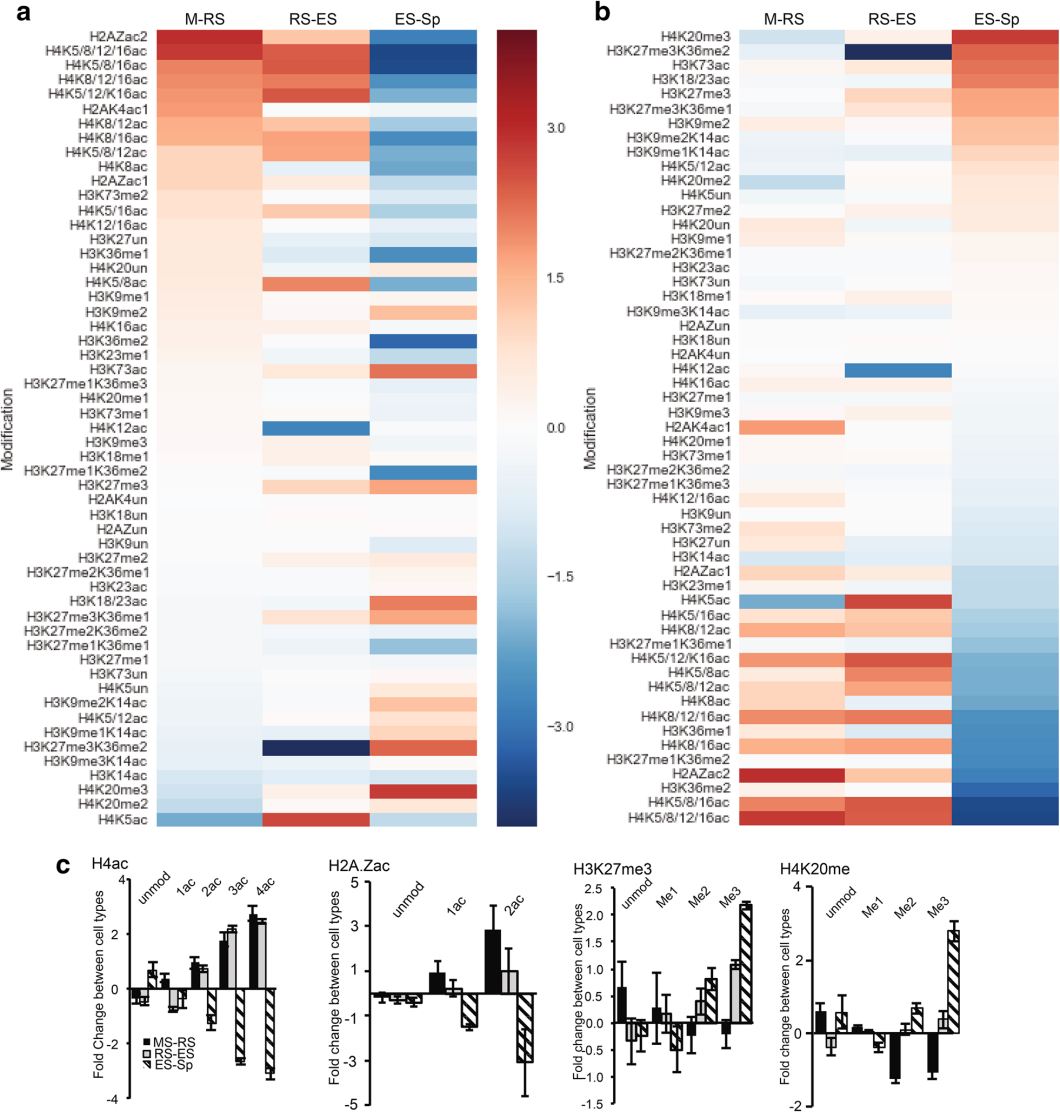
Dynamic changes in histone PTMs during mouse spermatogenesis. Heatmaps depicting fold changes of individual or combinatorial histone PTMs during sequential stages of spermatogenesis, i.e., meiotic to round spermatids (M-RS), round spermatids to elongating spermatids (RS-ES), and elongating spermatids to mature sperm (ES-Sp). Histone PTMs are ranked and ordered based on greatest fold increase in the M-RS transition (**a**) or greatest fold increase in ES to Sp (**b**). **c** Fold changes of acetylated lysine residues on the H4K5 peptide, H2A.ZA1 peptide, H3K27me3, and H4K20me during spermatogenesis

We next calculated the fold change of individual and combinatorial PTMs between subsequent stages of spermatogenesis and ranked them according to the relative abundance fold change from M to RS (Fig. 2a). We observed the most dynamic changes of relative histone PTM abundance from acetylation on histone H2A.Z and H4 (Fig. 2a). Combinatorial acetylation of the K4 and K15 residues on H2A.Z exhibited the greatest fold change (+2.94) during the initial stages of spermiogenesis (M to RS) before a decrease in the final ES to Sp stage (Fig. 2a). Histone acetylation dramatically increased on individual H4 lysine residues K8, K12, and K16 from M to RS and RS to ES prior to a dramatic loss of acetylated residues in Sp (Fig. 2a). In contrast, the relative abundance of H4K5ac decreased from M to RS (bottom, Fig. 2a). However, quantification of combinatorial modified peptides showed the most robust increase occurs from the M to RS transition when all four of the H4 lysine residues are acetylated (K5/8/12/16, top, Fig. 2a).

 While numerous modifications followed a pattern of increasing relative abundance from M to RS followed by a loss of modification in Sp, some PTMs showed a pattern of enrichment in the final stages of germ cell maturation (Fig. 2b, PTMs ranked by fold change during ES to Sp). For example, the repressive H3K27me3 modification showed a striking increase in relative abundance in the final stages of sperm maturation (RS to ES, EC to Sp, Fig. 2a, b; Additional file 1). Similarly, the H4K20 residue exhibited a dynamic change in methylation status during sperm maturation, beginning with an increase in monomethylation (H4K20me1) in M to RS, followed by a shift to di- and trimethylated forms in ES and Sp cells (Fig. 2a, b). Additionally, we observed a number of PTMs exhibiting distinct dynamics in relative abundance (Fig. 2a, b; Additional file 2).

### Identification of histone post-translational modifications in human sperm

There is a striking dearth of knowledge regarding the spectrum of histone PTMs present in human sperm. Therefore, we performed a similar analysis utilizing nano-LC-MS/MS on isolated histones in sperm cells from normozoospermic semen samples. Of specific interest is whether PTMs differ between individuals given the significant heterogeneity observed in a human semen sample. In particular, in any single sample, the vast majority of sperm have abnormal motility, progression, and morphology and 40–60 % of the sperm are fully immotile [27]. There are also significant differences in the composition of semen parameters between individuals; for example, in our samples, motility ranged between 41 and 66 % and progression 33 and 49 % (Table 2). We therefore aimed to investigate the presence and abundance of histones and PTMs in sperm from different individuals. As such, 8 normozoospermic semen samples were obtained from men undergoing routine semen analysis at the University of Pennsylvania Fertility Clinic. All samples met the criteria for normal semen analysis according to the WHO 5th edition [27] and strict Kruger parameters for normal morphology [30]. Semen characteristics are shown in Table 2.

**Table 2:** Semen parameters for individual samples including count, motility, progression and morphology

Sample number	Count (×10^6^)	Motility (%)	Progression (%)	Morphology (%normal)
1	65	58	41	6
2	57	48	33	4
3	87	66	47	4
4	54	45	41	5
5	52	58	49	6
6	51	58	40	6
7	29	55	38	5
8	55	41	33	4

Nano-LC-MS/MS identified the canonical histone H3 and H4 proteins, 23 histone isoforms of H1, H2A, H2B, and H3, and 103 histone modifications present in human sperm (Table 3). Twenty-nine amino acid residues on canonical histones H3 and H4 were modified, and 9 PTMs on these amino acids are reported for the first time in human sperm (H3K18me and K23me, H4S1ph, R3me, K31ac, R35me, M84ox, K91ac, R92me). A number of histone isoforms of H1, H2A, and H2B were identified in human sperm. These included four testes-specific histone isoforms (H1.T, H1.T2, TH2B, and H3.1T). On the histone isoforms of H1, 17 amino acid residues contained PTMs, and ten amino acid residues on H2A histone isoforms and 14 residues on H2B histone isoforms were modified. Finally, 15 amino acid residues on H3 histone isoforms contained modifications. Of note, all PTMs found on histone isoforms in human sperm are reported for the first time (Table 3). In addition to modifications such as lysine methylation and acetylation, several examples of crotonylation, oxidation, phosphorylation, and arginine methylation were also classified for the first time in human sperm.

**Table 3:** Histone isoforms and post-translational modifications (PTMs) identified in human sperm

*Histone isoforms*	
H1	H1.4, H1.t, H1.t2
H2A	H2A.1a, H2A.2b, H2A.2c, H2A.3, H2A.J, H2A.V, H2A.X, H2A-bbd 2/3, H2A.Z, macroH2A.1, macroH2A.2
H2B	tH2B., H2B.1b, H2B.1c/e/f, H2B.1d, H2B.1 l, H2B.2f
H3	H3, H3.3, H3.1t
H4	H4
*Histone post*-*translational modifications*	
H1	*K43ac, R50me1, K62me1, K63ac*
H1t	*K112me1, K113ac, K122ac, K124me1, K170ac, K173me1, K183ac, K183me3, R185me1, S180ph, S187ph, K188me1, K190ac*
H2A	*K5ac, R11me2, R29me1*
H2AV/Z	*K4ac, K4me2/3, K7ac, K7cr, K7me1/2/3, S9ph, K11cr, K11me1/2/3, K13cr, K13me2/3, K15ac, R19me1, K27ac, K37cr, K37me1*
H2B	*K16me1, K20me3*
tH2B	*K6ac, T9ph, K12ac, K12me1/3, K13me1/3, K16me1/3, K17me1, K28ac, K29ac, R30me2, K86ac, R87me1*
H3	T3ph, K4me1/2/3, K9ac, K9me1/2/3, K14ac, *K18ac, K18me1,* K23ac*, K23me1/3*, *R26me1/2*
	K27me1/2/3, *K36cr,* K36me1/2/3, *K37ac,* *K37me2/3, R53me1, K56ac,* K79me1/2, *K120Ox*
H4	*S1ph, R3m1e*, K5ac, K8ac, K12ac, K16ac, K20me1/2/3, *K31ac, R35me1, M84Ox, K91ac, R92me1*

Includes PTMs identified on any isoform except those with modified, non-conserved amino acid residues (H1t, H2AV, H2AZ, tH2B). PTMs in italics represent those reported for the first time in human sperm

### Relative abundance of histone PTMs in human sperm

Mass spectrometry was next used to compare the relative abundance of common histone modifications between individual human sperm samples. A label-free approach was used to calculate the relative abundance of the modified and unmodified forms of each specifically cleaved peptide. By calculating the area under the curve for each form, the relative abundance can be expressed as a percentage of the total peptide forms. On the H4 peptide encompassing amino acids 4-17 (aa4-17), the predominant form was unmodified (40.3 %), followed by mono- (28.9 %), di- (16.5 %), tri- (8.5 %), and quadruple acetylation (5.8 %). Among single PTM, H4K16ac was the most common form of the H4 peptide aa4-17 (22.5 %). Furthermore, among combinatorial modifications, H4K8acK12ac was most abundant (9.4 %) followed by K5acK8acK12ac (5.6 %) and K5acK8acK12acK16ac (6.3 %). For the remainder of the H4 tail peptide aa20-23, the dimethylated form of H4K20me2 was the most abundant form (80.9 %) followed by H4K20me3 (9.8 %) and H4K20me1 (7.9 %). Only a small portion of peptide H4aa20-23 was unmodified (1.4 %).

More detailed examination of H3 showed that H3K9me3 was in greatest abundance on peptide aa9-17 (27.9 %), followed closely by the unmodified peptide (26.9 %) and monomethylated (19.9 %) and dimethylated form (18.7 %). Notably, small amounts of H3K14ac were also detected (5.5 %) (Additional file 3). The most common PTMs on peptide aa27-40 were combinatorial H3K27me1/K36me2 (55.6 %). While H3K27me2 (7.5 %) and K27me3 (3.0 %) were both detected, monomethylated H3K27me1 was negligible, except in combination with K36me2/me3. This was also the case with H3K36, which was isolated in dimethylated form (4.3 %); however, the mono- and trimethylated forms were measurable only in combination with K27me/me2/me3. Notably, the unmodified peptide was not detected. Finally, for the peptide containing H3K79, the predominant form was H3K79me2 (57.4 %), followed by the unmodified peptide (33.2 %) and H3K79me1 (9.5 %).

### Uniformity of histone PTMs in individual semen samples

**Fig. 3:**
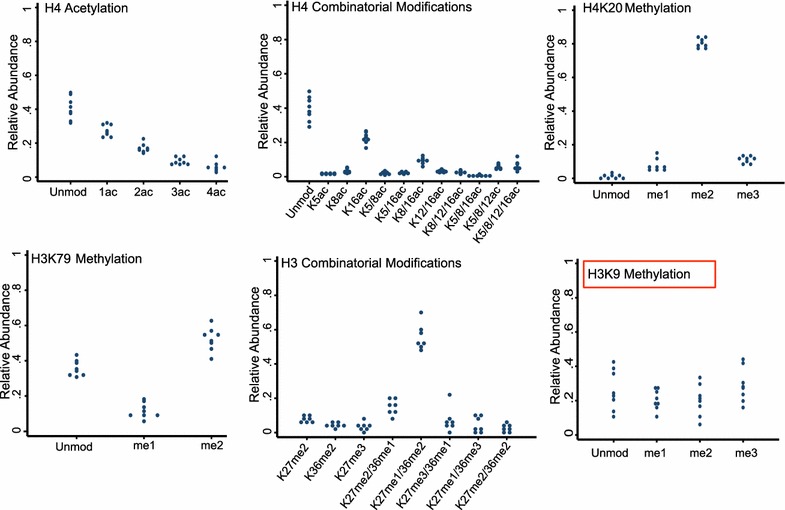
Relative abundance of histone PTMs on H4 and H3 in human sperm. Dotplot demonstrating relative abundance of individual and combinatorial PTMs on H4 and H3 in different individual sperm samples

The significant heterogeneity in semen parameters both within and between individual sperm samples prompted examination of the PTMs between different individuals. Strikingly, the relative abundance of histone modifications on H3 and H4 between individuals was similar (Fig. 3; Additional file 1). To quantify the variance between samples for each specific modification, the coefficient of variation (CV) was calculated by dividing the standard deviation by the mean for each modification with greater than 10 % overall abundance (Additional file 4). This confirmed the tight regulation of acetylation and methylation on H4, with maximum CV of 16.52. Similarly, modifications on histone H3 were also overall conserved, with a maximum CV of 29.46. The exception was methylation of the H3K9 peptide, which varied between individuals (CV of as much as 45.44).

### Conservation of histone PTMs in mouse and man

**Fig. 4:**
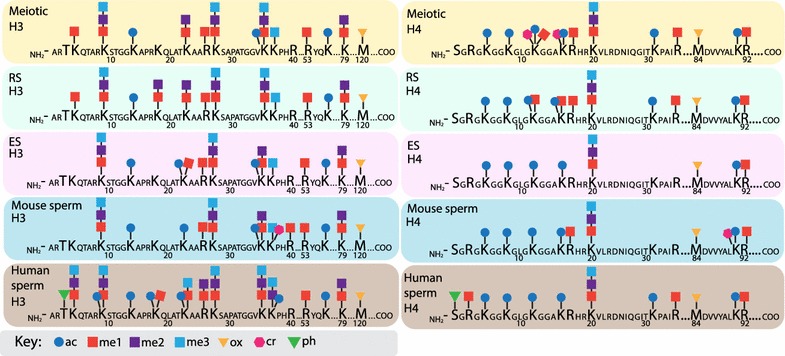
Schematic of histone H3 and H4 post-translational modifications (PTMs) in mouse and human male germ cells. PTMs included in this schematic were identified as present on any isoform through computational analysis. Key for each post-translational modification included in diagram

**Fig. 5:**
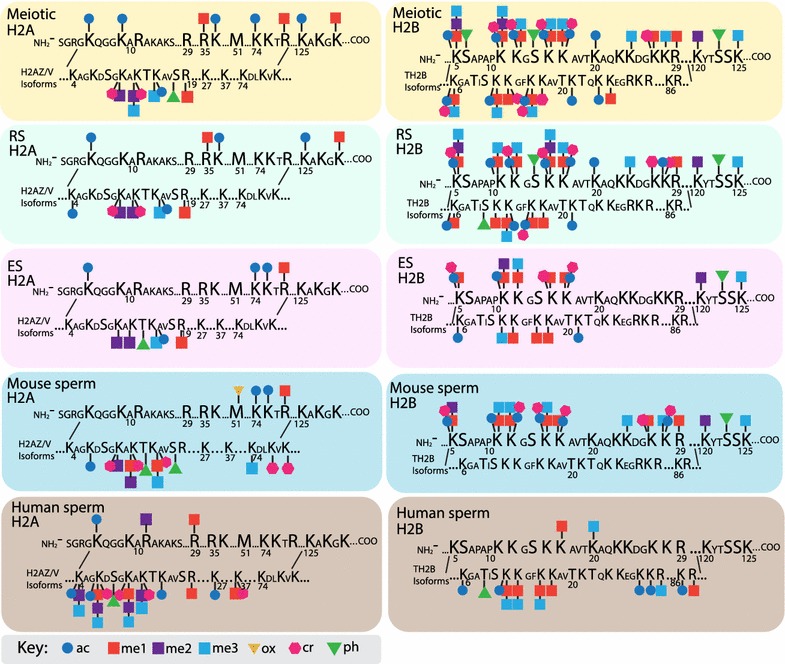
Schematic of histone H2A and H2B post-translational modifications (PTMs) in mouse and human male germ cells. PTMs included in this schematic were identified as present on any isoform through computational analysis. Non-conserved regions of histone isoforms H2AV, H2AZ, and tH2B are located below conserved sequence. Key for each post-translational modification included in diagram

We compared the PTM profiles of mature mouse sperm with human sperm to identify histone modifications conserved across species (Figs. 4, 5). Strong conservation of PTMs was detected on histones H3 (17/20 mouse PTMs shared with human) and H4 (10/12), including a number of modifications previously shown to play key roles in spermatogenesis and sperm function (H3K9me/2/3, K23ac, H3K27me/2/3, H3K36me/2, K79me/2, M120Ox, H4K5/8/12/16ac, H4K20Me/2/3, K91ac, R92me) [21–24, 31–34]. However, also striking is that very few histone PTMs were conserved between mouse and human on histones H2A (5 shared PTMs), H2B (1), and H1 (0). This result was somewhat unexpected and could potentially be due to the low abundance of these histone PTMs on the small amount of retained histones. Further validation and analysis are warranted to determine whether these PTMs are actually non-conserved between mice and humans. Certain similarities in relative abundance of specific PTMs also occur, including predominance of H4K20me2 in mature mouse and human sperm.

## Discussion

The covalent attachment of post-translational modifications to histones is a major epigenetic mechanism, linked to regulation of cellular processes during spermatogenesis and in mature sperm [1, 2, 35]. Numerous informative studies have investigated specific histone PTMs in male germ cells using antibody-based approaches, and recent studies have utilized mass spectrometry to identify histone PTMs in mature mouse sperm [25]. However, to date, a comprehensive ‘map’ and detailed investigation into histone PTMs present during multiples stages of spermatogenesis have been lacking. In this study, we used a “bottom-up” nano-LC-MS/MS methodology as an accurate, unbiased, and quantitative approach to identify histones, histone isoforms, and histone PTMs present in multiple stages of spermatogenesis and in mouse and human sperm. To our knowledge, this is the first comprehensive characterization of the histone PTM complement in immature male germ cells, thus providing an important resource that additionally affords a more complete understanding of the dynamic changes in these chromatin markers as mature spermatozoa are formed. Furthermore, we analyzed several clinically defined normal individual human semen samples to create a comprehensive snapshot of the histone PTMs present in human sperm, which surprisingly was highly consistent across individuals. Finally, we found a high level of histone PTM conservation between mouse and human sperm on histones H3 and H4, but surprisingly few conserved modifications on H2A and H2B. Together, the comprehensive analysis of histone PTMs in developing and mature mammalian sperm provides a crucial resource for continued investigation of the paternal epigenome.

Our analysis of the relative abundance of histone PTMs during multiple stages of spermatogenesis revealed that the most dynamic changes in histone PTMs occur through hyperacetylation of histone tails from meiosis through the round and elongating/condensing spermatid stages, followed by the subsequent loss of acetylated histones in mature sperm. This finding supports the widely held dogma that histone hyperacetylation is necessary for histone eviction [2, 20]. In our analyses, acetylation of H2A.Z exhibited the greatest increase from the M to RS stage (Fig. 2a, b). While incorporation of the H2A.Z variant was identified at transcriptional start sites in round spermatids [36], only histone H2A was hyperacetylated during spermiogenesis [19]. Histone H4 hyperacetylation has previously been detected in round and elongating spermatids at the K5, K8, K12, and K16 residues [19, 37]. Our analysis using nano-LC-MS/MS builds on these findings by quantitatively assessing the changes in relative abundance of each individual acetylated amino acid during the M to RS and RS to ES transition. We observe increases in acetylation of the individual K8, K12, and K16 residues from M to RS (Fig. 2a). H4K12ac then strongly decreases in the RS to ES stage before remaining lowly abundant in mature sperm (Fig. 2a; Table 1). This pattern suggests that histones with high H4K12ac may be rapidly evicted or replaced in the RS stage prior to marking retained histones in mature sperm [32–34]. In contrast, we see a distinct decrease in K5ac (Fig. 2a) from M to RS, prior to an increase in the RS to ES stage, thus suggesting that individual K5ac may be a key marker in a secondary wave of histone hyperacetylation. Intriguingly, the combination of multiple acetylated H4 residues (2Ac, 3Ac, or 4Ac) provides the greatest increase in hyperacetylation, indicating that acetylation of multiple residues on the same nucleosome may better facilitate the loosening of chromatin necessary for histone eviction. Future studies will be needed to examine functional relevance of nucleosomes with H4 tails bearing single PTM compared to those H4 histones bearing multiple PTMs.

Another PTM of interest that dynamically changes during spermatogenesis is H3K27me3, which exhibits a striking increase in abundance in the final stages of spermatogenesis and is enriched in mature sperm (ES to Sp). H3K27me3 is a repressive histone PTM [38, 39]; thus, enrichment on retained histones may be key in providing transcriptional silencing in mature sperm. H3 K27me3 located on the same nucleosome as the activating H3K4me3 PTM is termed a “bivalent signature” that is thought to poise genes for rapid transcriptional induction in embryonic stem cells following removal of H3K27me3 [40]. Our data supports the presence of H3K27me3 in meiosis, round spermatids, and the enrichment in mature sperm suggests that H3K27me3 may mark histones for retention. Unfortunately, our nano-LC-MS/MS analysis was unable to consistently detect the previously identified H3K4me3 [21, 24, 41] in mouse germ cells due to low PTM abundance and short retention time in mammalian cells [42].

Importantly, our method of mouse germ cell collection (STAPUT velocity sedimentation [28]) represents a continuum across each cell stage. For instance, our meiotic cell population encompasses primary and secondary spermatocytes at all stages of meiosis. Additionally, we did not detect certain histone isoforms and PTMs previously identified in male germ cells, including multiple PTMs on the spermatid-specific linker histone H1-like protein (HILS) [43]. While we detected the HILS protein in mouse testes, we did not observe PTMs; this is likely due to an overall small proportion of the histone isoform in the acid-extracted protein pool. To better characterize such variants, it will be necessary to enrich for the histone isoform or specific PTM prior to nano-LC-MS/MS, as was reported for HILS [43]. Additionally, as the “bottom-up” nano-LC-MS/MS methodology utilized in this study is dependent upon the cleavage of peptides by trypsin, it is important to note that it is not possible to distinguish between short peptide sequences of homologous histone isoforms (i.e., portions of H3.1 and 3.3) and associated PTMs. Additional analysis utilizing other strategies such as top-down LC-MS/MS is necessary to definitively identify and quantify highly homologous histone isoforms and variants that differ by only a few amino acids.

Using our nano-LC-MS/MS method, we identified a total of 77 different histone PTMs across H1, H2A, H2B, H3, and H4 in mature mouse sperm from SV129 mice. This is dramatically higher than previous mass spectrometry analyses of C57BL/6 mouse sperm, which identified 26 histone PTMs, and none were located on histone H2A [25]. Our analysis identified a total of 18 PTMs on the multiple H2A isoforms. This large increase in the number of identified PTMs could be due to the high sensitivity of our nano-LC-MS/MS instrumentation, different analytical methods, or strain-specific differences.

Our mass spectrometry analysis in human sperm revealed a number of histone and testes-specific histone isoforms, consistent with prior proteomic analyses of human sperm [44, 45]. We were also able to comprehensively assess individual and combinatorial PTMs, among which some are described for the first time in human sperm. These include classic PTMs such as methylation and acetylation as well as more recently described PTMs, such as crotonylation. Interestingly, histone crotonylation was found to mark testes-specific genes that become post-meiotically activated in the mouse, thereby escaping sex chromosome inactivation [46]. We have now confirmed the presence of crotonylation in both mouse and human mature sperm.

Importantly, we were also able to quantify the abundance of specific PTMs between individual normozoospermic semen samples. The establishment of a histone PTM profile of normozoospermic sperm is a first step in the further investigation of important clinical and translations questions regarding possible alterations to these profiles in the setting of clinical pathology such as sperm abnormalities, malignancy, or environmental influences. Unfortunately, as the sperm analyzed in this study were obtained from discarded and de-identified semen, we were unable to assess the reason for semen analysis. Therefore, it is possible that while semen samples were classified as normozoospermic, they may still have come from men who are part of a couple with infertility. Nonetheless, we detected a remarkable consistency in the relative abundance of histone PTMs on H3 and H4 in these normozoospermic samples.

Prior analysis of histones in human sperm has largely focused on the overall localization of retained nucleosomes and selected PTMs. Previous studies of specific PTMs have focused on modifications such as H3K27me3 and H3K4me2/3, which were found to be enriched at developmental promoters and signaling transcription factors [21, 23]. Other PTMs such as H4K12ac have also been described at promoters of genes involved in developmental processes and CTCF-binding sites [32]. While the overall localization of retained nucleosomes is currently a subject of debate, the conserved packaging and marking of histones is hypothesized to represent both a historical record of spermatogenesis while also potentially playing an important role in early preimplantation processes or developmental programming [3, 47]. While our approach does not provide information regarding the localization of histones in male germ cells, it does lend support to this hypothesis as we found that the relative abundance of specific histone PTMs on H3 and H4 is overall highly consistent between different individual humans. Given the significant heterogeneity both within and between sperm samples, this striking consistency supports a process of programmatic retention of specifically modified histones. Of note, we did see variation in the abundance of H3K9me between different individuals. Interestingly, analysis of human B cells identified genetic variation in the expression of KDM4C, which corresponded to differences in the ratio of H3K9me3/2 in these human cells [48].

Our findings that H3K9me3 and H4K20me3 are conserved between the mouse and human lend further support to an evolutionarily conserved role of these PTMs in proper repression of paternal heterochromatin. Recently, H3K9me3 and H4K20me3, two well-defined markers of heterochromatin, were shown to be transmitted by the sperm to the embryo and present an intergenerational model for constitutive heterochromatin in the early human embryo [31]. Micrococcal nuclease digestion (MNase) followed by next-generation sequencing has also associated histone localization with centromere repeats and retrotransposons (LINE1 and SINEs) in human sperm [47]. While no specific histone PTMs were analyzed in relation to LINE1 and SINE elements, it would be interesting to determine whether H3K9me3 and H4K20me3 mark enriched histones in these elements. Of note, analysis of our mouse and human data sets for the centromeric specific H3 isoform CENP-A did not yield any definitive identification. This is likely due to the low abundance of this specific histone isoform, necessitating enrichment-based methodologies to positively detect CENP-A in male germ cells by nano-LC-MS/MS [49].

The accumulating evidence suggests a role for retained histones and PTMs in early development and programming; we further hypothesize that the balance of specifically marked histones may also play a role in normal fertility. Indeed, several studies have begun to examine the relationship between histones and human fertility. Prior work demonstrated the importance of proper histone to protamine exchange, with abnormal protamination associated with male infertility [7, 8, 50]. Furthermore, decreased hyperacetylation has been described on H4 in men with decreased fertility and impaired spermatogenesis [51]. It has also been shown that men from couples with infertility display a more dispersed histone pattern and have an overall reduction in the amount of H3K4me and H3K27me retained at developmental transcription factors and certain imprinted genes [22]. Shifts in the distribution of H3K9ac have also been described in the sperm of infertile men [52], further suggesting the potential of epigenetic regulation of the paternal genome.

## Conclusions

These emerging data highlight the need for additional research examining how PTMs may impact normal fertility. Our data revealing the presence and consistency of specific PTMs furthers current appreciation of the importance of appropriate histone PTMs in normal human sperm and provides important insight into conserved PTMs in the mouse. Coupled with our analysis of dynamic changes in PTMs during mouse spermatogenesis, these data provide an important resource for translational studies of infertility. Future studies exploring how this complement is altered in the setting of impaired fertility in mice and humans are needed to provide insight into the functional significance of these normal histone PTM levels.

## Methods

### Collection of mouse germ cells

Sexually mature, adult male SV129 mice (8–12 wks old, Charles River Laboratories) were ethically and humanely euthanized by CO_2_ asphyxiation as approved by the University of Pennsylvania Institutional Animal Care and Use Committee. Immature male germ cells were collected from testes by STAPUT velocity sedimentation as previously optimized and described by our laboratory [28]. Briefly, for each STAPUT collection testes from 11 adult SV129 mice were dissociated to a single cell suspension with sequential incubation of collagenase (0.9 mg/ml) and trypsin (0.6 mg/ml) with DNase (0.1ug/ml) in KREBs buffer. Testes cells were subsequently resuspended in 0.5 % BSA and slowly loaded onto a 2–4 % BSA gradient. Cells were allowed to sediment for 105 min prior to fraction collection. Cell aliquots were stained with DAPI and assessed by fluorescent microscopy for meiotic (M, ~1.5 × 10^7^ cells/STAPUT), round spermatid (RS, ~10^8^ cells/STAPUT), and elongating/condensing spermatid (ES, ~10^8^ cells/STAPUT) fractions (Fig. 1a; Additional file 5). Cell fractions with at least 85 % purity for each stage were pooled, snap frozen, and stored at -80 °C for further processing. Of note, the majority of contaminating cells in the ES fraction were composed of nuclear-free red blood cells. Mature sperm were collected from the cauda epididymis and incubated in somatic cell lysis buffer (0.1 % SDS, 0.5 % Triton-X-100) for 15 min to remove any non-sperm contaminants. Purity of sperm samples was confirmed through microscopic visualization. Pyrosequencing analysis to assess DNA methylation status for the paternally imprinted H19 allele (94 %) and maternally imprinted SNRPN allele (6 %) was consistent with previous reports for baseline levels of paternal and maternal allele methylation in sperm (Additional file 5 [53, 54]). For each collection the sperm of 11 animals were pooled, counted (~2x10^8^ sperm/collection), and subsequently snap frozen and stored at −80 °C until further processing. To allow for biological replication of experiments, three distinct STAPUT collections were performed (11 mice/collection, total of 33 animals) on adult SV129 male mice procured on three separate dates.

### Collection and processing of human samples

Discarded semen samples were obtained from the University of Pennsylvania Fertility Clinic from all men presenting for routine semen analysis. All samples were de-identified prior to processing; therefore, the clinical indication for semen analysis was unknown. As all samples were discarded and de-identified, the University of Pennsylvania Institutional Review Board determined this study was exempt from requiring informed consent. Ethical approval for this study was obtained from the University of Pennsylvania Institutional Review Board (protocol #815929). Following 2–5 days of abstinence semen samples were collected in a sterile container and assessed by trained andrology staff according to the WHO reference values, 5th edition [27] and strict Kruger criteria for morphology [30]. Samples, which met all of the following criteria, were considered as normal: semen volume ≥1.5 mL, sperm count ≥15 million/mL, motility ≥40 %, forward progression ≥32 %, and morphology ≥4 %. Semen samples were washed with PBS to remove seminal fluid. The resulting sperm were somatic cell lysed (0.1 % SDS, 0.5 % Triton-X-100) for 30 min on ice, microscopically inspected to ensure purity, counted, and stored at −80 °C until further use.

### Acid extraction, propionylation, and trypsin digestion of histones

Acid extraction of histones from all germ cell types for nano-LC-MS/MS analysis was conducted as previously described with minor modifications [26, 55]. Briefly, mouse and human sperm samples were treated with DTT (50 mM) for 30 min to aid in nuclear decondensation before immediately proceeding to hypotonic lysis. Male germ cells (M, 10^7^; RS, 2 × 10^7^; ES, 4 × 10^7^; Sp—2–5 × 10^7^) were rotated at 4 °C for 30 min in hypotonic lysis buffer (10 mM Tris–HCl pH 8.0, 1 mM KCl, 1.5 mM MgCl2, 1 mM DTT with protease inhibitors). Pelleted nuclei were subsequently resuspended in 0.4 M sulfuric acid and rotated overnight at 4 °C. The resulting supernatant was precipitated with 33 % trichloroacetic acid (TCA), incubated on ice for 2 h, and the subsequent histone pellet was washed twice with cold acetone prior to resuspension in DNase/RNase free water. Acid-extracted protein (1ug) was visualized by Coommassie-stained 12 % SDS-PAGE gels to determine sample quality (Fig. 1b, histones denoted by red box, and other acid-soluble protein extracts are observed in each lane). To prepare acid-extracted histones for nano-LC–MS/MS analysis, 15 ug of acid-extracted protein was used for propionylation and trypsin digestion. Each sample was treated twice with propionylation reagent (1 part propionic anhydride (Sigma), 3 parts 2-propanol) at pH 8 to block the ɛ-amino groups of unmodified and monomethyl lysine residues, allowing trypsin to perform proteolysis only at the C-terminal of arginine residues. Peptides were trypsin digested with 0.75ug trypsin (1:20 trypsin/protein ratio, Invitrogen) for 6 h at 37 °C and subsequently treated with two additional rounds of propionylation. The resulting propionylated and trypsin-digested peptides were stage-tip desalted with C18 mini-disks and stored at −80 °C until nano-LC-MS/MS analysis (Additional file 1).

### Tandem mass spectrometry

The resulting acid-extracted, propionylated, and trypsin-digested histone peptides were purified with C18 stage-tip for mass spectrometry analysis. Desalted histone peptides were then loaded onto and separated by reversed-phase HPLC on a Thermo Scientific™ EASY-nLC 1000 system with a 75-um i.d. × 15 cm ReproSil-Pur C18-AQ 3 μm nanocolumn run at 300 nL/min. Peptides were eluted with a gradient from 2 to 30 % ACN (35 min) and to 98 % ACN over 20 min in 0.1 % formic acid. For mouse histone samples, the HPLC was coupled to a Thermo Scientific™ Orbitrap Elite™ Hybrid Ion Trap-Orbitrap Mass Spectrometer. In each cycle, one full MS Orbitrap detection was performed with the scan range of 290 to 1400 *m/z*, a resolution of 60 K, and AGC of 1 × e6. Then, data-dependent acquisition mode was applied with a dynamic exclusion of 30 s. Ten MS2 scans were followed on parent ions from the most intense ones. Ions with a charge state of one were excluded from MS/MS. An isolation window of 3 *m/z* was used. Ions were fragmented using collision-induced dissociation (CID) with collision energy of 35 %. Ion trap detection was used with normal scan range mode and normal scan rate. The resolution was set to be 15 K with AGC of 1 × e4. For human sperm histone samples, the HPLC was coupled to a Thermo Scientific™ Q Exactive™ Hybrid Quadrupole-Orbitrap Mass Spectrometer. In each cycle, one full MS Orbitrap detection was performed with the scan range of 290 to 1600 *m/z*, a resolution of 70 K, and AGC of 1 × e6. Then, data-dependent acquisition mode was applied with a dynamic exclusion of 30 s. MS2 scans were followed on parent ions from the most intense ones. Ions with a charge state of one were excluded from MS/MS. An isolation window of 3 *m/z* was used. Ions were fragmented using higher-energy collisional dissociation (HCD) with collision energy of 24. The resolution was set to be 17.5 K with AGC of 1 × e5. Targeted scans were performed on a number of peptides to increase the identification of low-abundance modifications. Histone PTM quantification was performed by using both in-house developed software EpiProfile [56] and manual verification.

### Peptide identification and quantification

A histone fasta file is built from the UniProt human or mouse database, including H1, H2A, H2B, H3, and H4. Raw files are searched with Mascot in Proteome Discoverer with the following search parameters: precursor mass tolerance ±10 ppm, fragment mass tolerance ±0.02 Da for HCD and 0.5 Da for CID, trypsin only cleaving after arginine and up to two miscleavages, peptide N-terminal propionylation (Propionyl[Peptide N-term]/+ 56.026) as the fixed modification. To obtain more identification for different kinds of peptides, we set the following variable modifications: (1) Propionyl[K]/+ 56.026 for unmodified peptides, (2) Acetyl[K]/+ 42.011 for acetylated peptides,(3) Methyl_Propionyl[K]/+ 70.042 for monomethylated peptides, (4) Dimethyl[K]/+ 28.031 for dimethylated peptides, (5) Trimethyl[K]/+ 42.047 for trimethylated peptides, (6) Phospho[ST]/+ 79.966 for phosphorylated peptides, (7) Crotonyl [K]/+ 68.026 for crotonylation peptides, and (8) oxidation[M]/+ 15.995 for methionine oxidation peptides. The target-decoy approach was used to filter the search results, in which the false discovery rate was less than 1 % at the spectral level [57].

The relative abundance of each modified and unmodified peptide was determined by measuring the area under the curve for each peptide form (unmodified or modified) based on MS1 spectra. Next, the total abundance of each peptide was calculated based on the sum of all unmodified and modified peptide forms. The relative abundance of each specific modified or unmodified peptide form was determined by dividing the area under the curve by total peptide abundance and multiplying by 100 to determine the percent of total peptide. For each peptide quantified, the total relative abundance equals 100 %. The average PTM standard deviation of the relative abundance for each cell type in the mouse testis was as follows: meiotic (0.61 %), RS (1.23 %), ES (1.08 %), and Sp (1.75 %).

## Additional files

10.1186/s13072-016-0072-6 Heatmap depicting fold-changes of individual or combinatorial histone PTMs during sequential stages of spermatogenesis, i.e., meiotic to rounds spermatids (M-RS), round spermatids to elongating spermatids (RS-ES), and elongating spermatids to mature sperm (ES-Sp). Histone PTMs are ranked and ordered based on greatest fold increase in the ES to Sp transition by histone.
10.1186/s13072-016-0072-6 Relative abundance of additional histone PTMs on H3 in human sperm. Dotplot demonstrating relative abundance of individual and combinatorial PTMs on H3 in different individual sperm samples.
10.1186/s13072-016-0072-6 Variation in PTM abundance between individuals. Coefficient of variation for histone modifications on H3 and H4 with a total abundance >10 %.
10.1186/s13072-016-0072-6 DAPI staining of mouse testis germ cell fractions. Representative images of final pooled STAPUT fraction from WT male mice. Left: meiotic fraction, Center: round spermatid fraction, Right: Elongating and condensing fraction. Bottom: Pyrosequencing analysis of DNA methylation in mouse sperm. Average percent of CpGs methylated in paternally imprinted H19 and maternally imprinted Snrpn (±SD).
10.1186/s13072-016-0072-6 Western blot validation of histone PTM analyzed by LC-MS/MS. Left: Acid extracted protein from male mouse germ cells exhibit similar levels of H3K27me3 and H4ac. Right Top: Western blot and coomassie stained protein gel of acid extracted histones from human sperm depicting variation in H3K9me3 between individuals, but not H4K20me2. Right Bottom: Western validation of H4K16ac in human sperm.

## Abbreviations

PTM: post-translational modification
Nano-LC–MS/MS: nanoliquid chromatography–tandem mass spectrometry
ART: assisted reproductive technologies
IVF: in vitro fertilization
ICSI: intracytoplasmic sperm injection
ph: phosphorylation
ac: acetylation
me: methylation
cr: crotonylation
WHO: World Health Organization
M: meiotic
RS: round spermatid
ES: elongating/condensing spermatid
Sp: mature sperm
CV: coefficient of variation
