# Supplementary figures and images for: Comprehensive analysis of histone post-translational modifications in mouse and human male germ cells
Source: Epigenetics Chromatin. 2016 Jun 21;9:24. doi: 10.1186/s13072-016-0072-6 (PMC4915177; doi:10.1186/s13072-016-0072-6)

Additional file 1

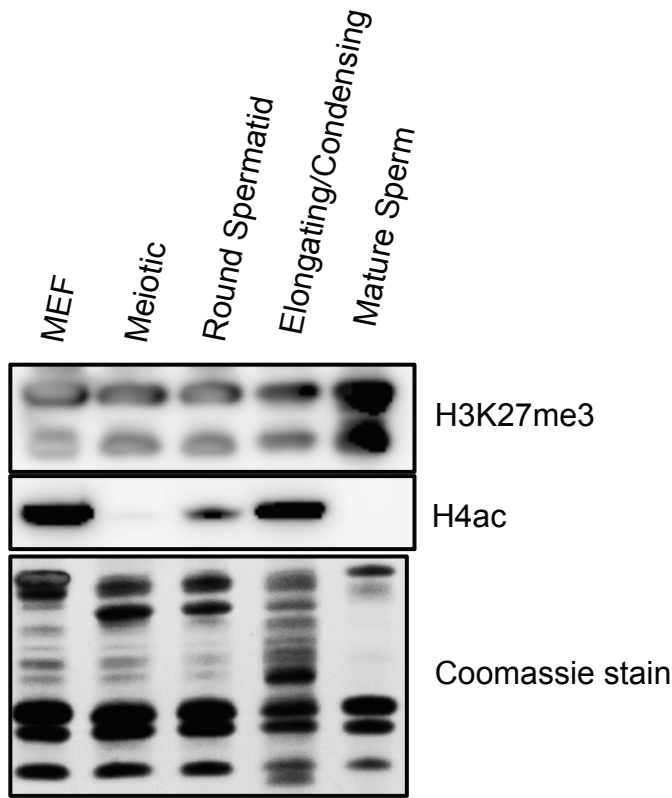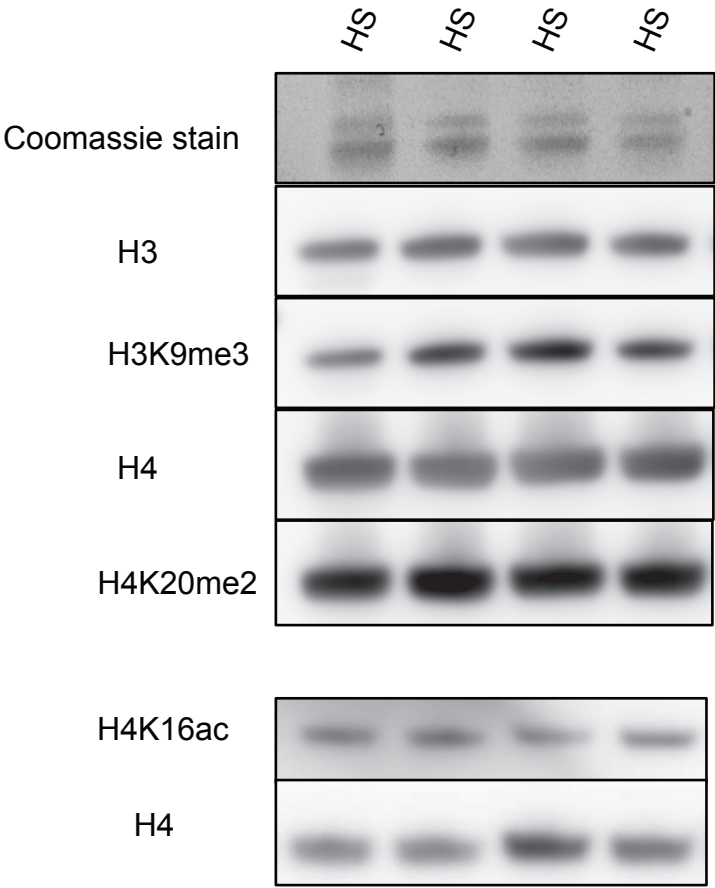

Supplement: Supplementary file 1 — 10.1186/s13072-016-0072-6 Heatmap depicting fold-changes of individual or combinatorial histone PTMs during sequential stages of spermatogenesis, i.e., meiotic to rounds spermatids (M-RS), round spermatids to elongating spermatids (RS-ES), and elongating spermatids to mature sperm (ES-Sp). Histone PTMs are ranked and ordered based on greatest fold increase in the ES to Sp transition by histone. [file 13072_2016_72_MOESM1_ESM.pdf]

# Additional file 2

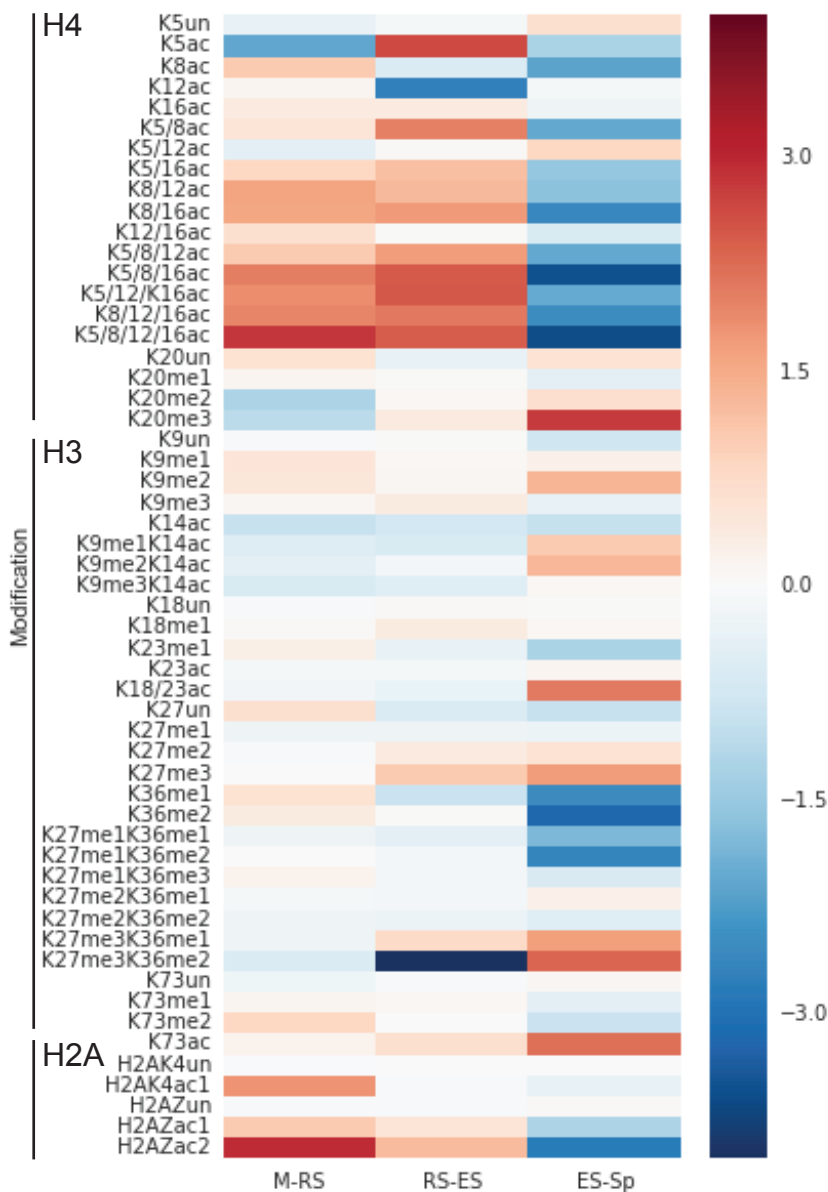

Supplement: Supplementary file 2 — 10.1186/s13072-016-0072-6 Relative abundance of additional histone PTMs on H3 in human sperm. Dotplot demonstrating relative abundance of individual and combinatorial PTMs on H3 in different individual sperm samples. [file 13072_2016_72_MOESM2_ESM.pdf]

# Additional file 3

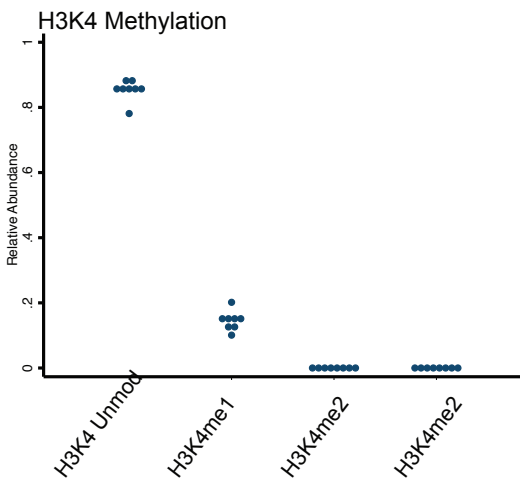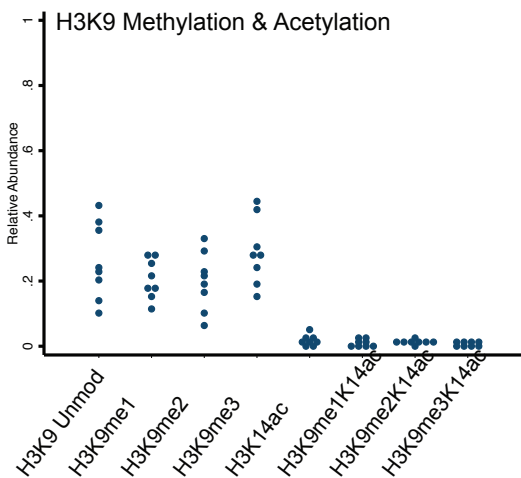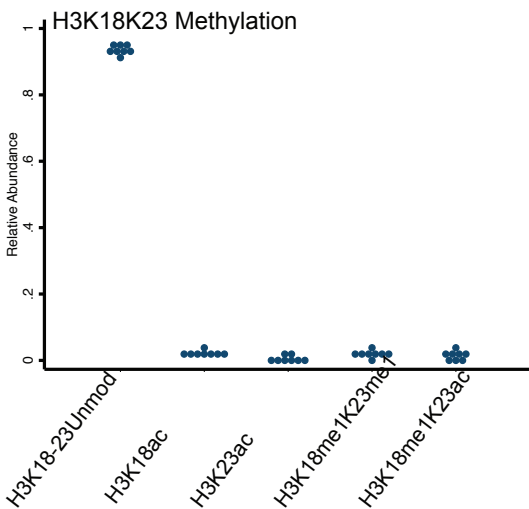

Supplement: Supplementary file 3 — 10.1186/s13072-016-0072-6 Variation in PTM abundance between individuals. Coefficient of variation for histone modifications on H3 and H4 with a total abundance >10 %. [file 13072_2016_72_MOESM3_ESM.pdf]

Additional file 5

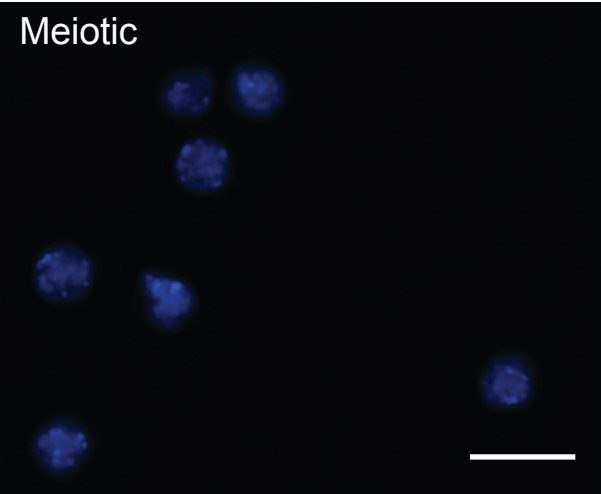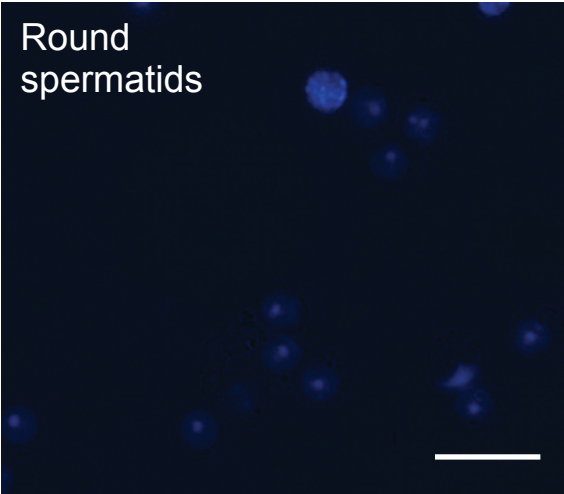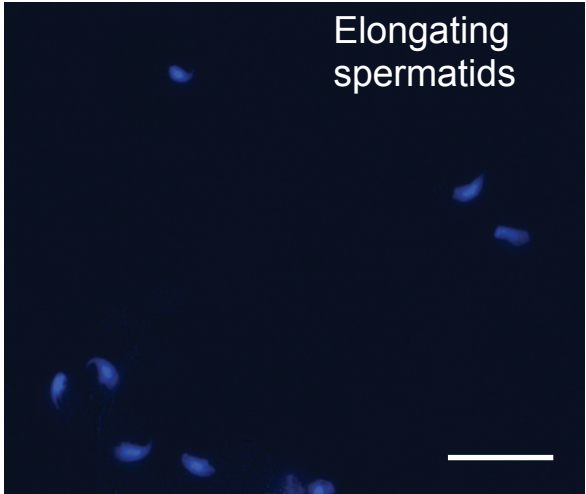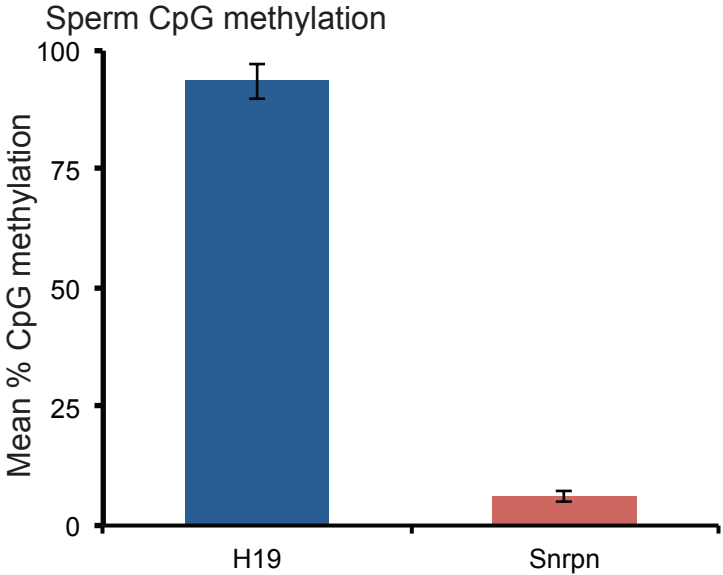

Supplement: Supplementary file 5 — 10.1186/s13072-016-0072-6 Western blot validation of histone PTM analyzed by LC-MS/MS. Left: Acid extracted protein from male mouse germ cells exhibit similar levels of H3K27me3 and H4ac. Right Top: Western blot and coomassie stained protein gel of acid extracted histones from human sperm depicting variation in H3K9me3 between individuals, but not H4K20me2. Right Bottom: Western validation of H4K16ac in human sperm. [file 13072_2016_72_MOESM5_ESM.pdf]
