# Supplementary material for: Comprehensive analysis of histone post-translational modifications in mouse and human male germ cells
Source: Epigenetics Chromatin. 2016 Jun 21;9:24. doi: 10.1186/s13072-016-0072-6 (PMC4915177; doi:10.1186/s13072-016-0072-6)
Supplement: Supplementary file 4 — 10.1186/s13072-016-0072-6 DAPI staining of mouse testis germ cell fractions. Representative images of final pooled STAPUT fraction from WT male mice. Left: meiotic fraction, Center: round spermatid fraction, Right: Elongating and condensing fraction. Bottom: Pyrosequencing analysis of DNA methylation in mouse sperm. Average percent of CpGs methylated in paternally imprinted H19 and maternally imprinted Snrpn (±SD). [file 13072_2016_72_MOESM4_ESM.docx]

**Additional file 4.** Coefficient of Variation (CV) for histone modifications on H3 and H4 with a total abundance of >10%.

**H3 Modifications CV H4 Modifications CV**

K4Unmod 3.33 H4Unmod 16.52

K4me1 20.75 H4Ac 12.95

K9K14Unmod 45.28 H42Ac 14.90

K9me1 28.83 H4K20me2 3.69

K9me2 45.44

K9me3 34.39

K18K23Unmod 1.65

K27me2K36me1 29.46

K27me1K36me2 12.78

K79Unmod 12.92

K79me2 12.56
